# Enamel maturation: a brief background with implications for some enamel dysplasias

**DOI:** 10.3389/fphys.2014.00388

**Published:** 2014-10-08

**Authors:** Colin Robinson

**Affiliations:** Department of Oral Biology, The Dental Institute, University of LeedsLeeds, UK

**Keywords:** enamel maturation, matrix protein loss, Porosity, uptake of foreign materials, enamel dysplasias

## Abstract

The maturation stage of enamel development begins once the final tissue thickness has been laid down. Maturation includes an initial transitional pre-stage during which morphology and function of the enamel organ cells change. When this is complete, maturation proper begins. Fully functional maturation stage cells are concerned with final proteolytic degradation and removal of secretory matrix components which are replaced by tissue fluid. Crystals, initiated during the secretory stage, then grow replacing the tissue fluid. Crystals grow in both width and thickness until crystals abut each other occupying most of the tissue volume i.e. full maturation. If this is not complete at eruption, a further post eruptive maturation can occur via mineral ions from the saliva. During maturation calcium and phosphate enter the tissue to facilitate crystal growth. Whether transport is entirely active or not is unclear. Ion transport is also not unidirectional and phosphate, for example, can diffuse out again especially during transition and early maturation. Fluoride and magnesium, selectively taken up at this stage can also diffuse both in an out of the tissue. Crystal growth can be compromised by excessive fluoride and by ingress of other exogenous molecules such as albumin and tetracycline. This may be exacerbated by the relatively long duration of this stage, 10 days or so in a rat incisor and up to several years in human teeth rendering this stage particularly vulnerable to ingress of foreign materials, incompletely mature enamel being the result.

## Definition of maturation stage

There is a very extensive literature on enamel development. With regard to the maturation stage there is a considerable amount of contemporary work dealing with cell physiology of the enamel organ and the molecular biology of the enamel matrix, but less so with the chemistry of the tissue. The following is intended to provide a brief basic background for this developmental stage with particular focus on tissue chemistry.

Enamel development has been divided into a series of consecutive stages based on tissue structure, histology of adjacent enamel organ cells and enamel chemistry (Warshawsky et al., 1981; Boyde, 1989; Robinson et al., 1995; Smith and Nanci, 1995).

Two stages can be most easily discerned. These are the secretory stage and the maturation stage. The secretory stage essentially entails the secretion of matrix and the initial mineral phase and can be considered complete when full thickness of the tissue has been laid down (Skobe, 1976).

This is followed by a maturation stage during which final matrix removal occurs and final mineral content is acquired. Final mineralization is probably completed post eruption, a phase not mediated by cells of the enamel organ (Robinson et al., 1997; Smith, 1998).

Between secretion and maturation there is a so called transition stage (Warshawsky and Smith, 1974; Robinson et al., 1981a). Since this appears to occur at or after major amelogenin secretion, it is included here essentially as an early phase of maturation. Many changes characteristic of maturation begin in transition and occur across the boundary between transition and maturation proper.

While these stages are fairly discrete, overlap with respect to certain functions does occur and will be mentioned where appropriate. While cellular, histological and chemical changes are similar in all species so far examined, the duration of this phase varies greatly from species to species.

## Histology of the enamel organ

After final enamel thickness has been achieved, enamel organ histology changes dramatically as the tissue moves into the transition stage (Warshawsky et al., 1981; Smith and Nanci, 1995).

### Transition

The transition stage involves reduction of the stratum intermedium to a single cell layer while fenestrated capillaries invade the enamel organ to approach the stratum intermedium layer (Garant and Gillespie, 2005).

The ameloblasts themselves, tall columnar cells about 70 μm in height reduce in height by about half. In addition, the long Tomes process through which the enamel secreted is reduced and is eventually lost (Reith, 1970). Internal structure of the ameloblasts also reorganizes. In brief, the nucleus takes up a more central position and the hitherto dense linearly arranged endoplasmic reticulum acquires a more disordered appearance. A comparison of histology with the appearance and chemistry of adjacent enamel has indicated that ameloblast shortening is complete at the beginning of maturation proper (Robinson et al., 1981a).

### Maturation

Maturation ameloblasts, now about 50% shorter than secretory cells, develop a rhythmic cyclic change in the membrane structure facing the enamel, from a smooth to a ruffled border (Josephsen and Fejerskov, 1977; Sasaki, 1984). This modulates in such a way that waves of ruffled membrane sweep along the maturing enamel toward the mature tissue. Cell-cell contacts also modulate. Ruffled bordered cells connect via tight junctions at the enamel surface. After loss of the ruffled border the tight junction is also lost (Inai et al., 2008). The result of these changes is that, particularly at the junction between transition and maturation proper, there is relatively free diffusible access to the enamel surface passing between ruffle ended cells then laterally between smooth ended cells into the enamel (Takano and Ozawa, 1984). This access has considerable implications for the access of foreign materials such as fluoride (Weatherell et al., 1975, 1977) and plasma proteins (Strawich and Glimcher, 1989; Robinson et al., 1992) into the tissue (see below) (see also Hammarström, 1967).

These morphological alterations reflect dramatically changed ameloblast function once the full thickness of the tissue has been laid down.

## Chemistry of enamel

### Transition/maturation

During transition, secretion of partially mineralized matrix stops as the surface limit of the tissue is reached and the processes associated with final mineral uptake begin.

## Organic matrix

Primary amelogenin ceases to appear at this stage (Robinson et al., 1983; Nanci et al., 1998; Wakida et al., 1999), and the enamel no longer increases in thickness. It is presumed that other matrix components, ameloblastin, enamelin, follow the same pattern. While there is evidence that some structural matrix proteins continue to be secreted during maturation (Smith and Nanci, 1996) the amounts appear to be barely detectable and their function, if any at this stage, is unknown. More obviously dramatic loss of organic matrix becomes apparent at this stage with about half of the matrix removed half way through maturation (**Figure 3C**).

**Figure 1:**
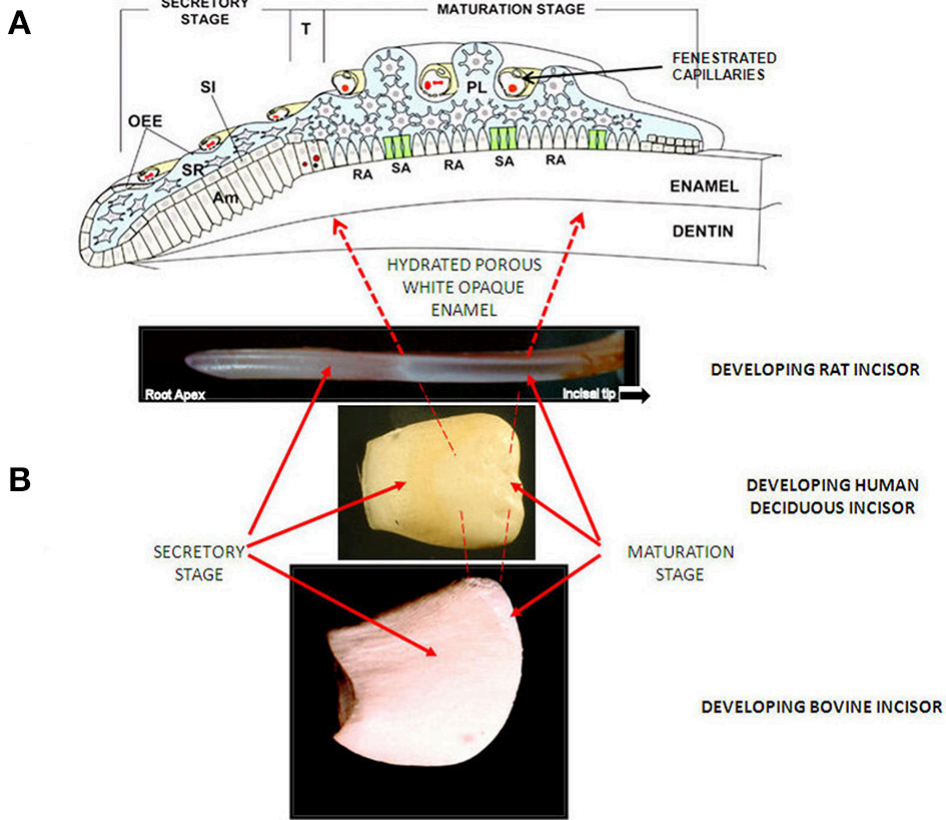
**(A)** Diagram of enamel organ at each stage of development. OEE; Outer Enamel Epithelium, SI; Stratum Intermedium, SR; Stellate Reticululm, Am; ameloblasts. T; Transition Stage, RA; Ruffle Ended ameloblasts, SA; Smooth Ended ameloblasts, P; papillary layer. With permission, modified from: Josephsen et al. (2010). *Am. J. Physiol. Cell Physiol*. 1299–1307. **(B)** Images of developing incisors from rat, human and cow, showing appearance (after drying) and position of secretory and maturation sages.

Importantly, matrix degradation products are replaced by fluid at transition/maturation (Hiller et al., 1975; Robinson et al., 1988) producing highly hydrated and porous tissue. After tooth extraction and some drying out, the porosity of the maturation stage is seen as a characteristic white opaque zone. (Figures 1B, **3A**).

Preceding matrix protein loss, proteolytic degradation occurs apparently following a two stage pattern. During secretion, an orchestrated degradation to specific components occurs via the enzyme MMP20, particularly of amelogenin (Brookes et al., 1995; Lu et al., 2008). During transition/maturation a second degradation phase begins mediated by the serine protease Kallikrein 4 (Lu et al., 2008). During this stage, the peptides produced by MMP20 are further degraded, much more completely. Kallikrein 4 activity initially appears at the end of the secretory stage but becomes most effective during transition and maturation. This results in the formation of small peptides and amino acids most of which disappear from the tissue (Brookes et al., 1995).

Small peptides and amino acids are presumably removed by the ameloblasts/enamel organ. Whether they diffuse out of the tissue or are removed actively is not clear. There is an argument that ruffle ended cells may carry out this function, because small organic molecules have been identified in ruffle ended cells. There is an intact basal lamina between these cells and the enamel so that any diffusion would be by lateral movement between smooth ended cells (Smith, 1998). Organic material remaining in maturing/mature tissue comprises an abundance of glycine and one or two tripetides (Glimcher et al., 1964; Weidmann and Hamm, 1965).

The amino acids and peptide fragments which remain are most likely bound to enamel crystals, for example, aspartate via anionic binding to crystals or glycine, the most dominant mature enamel species, which seems to fit into a spiral groove on apatite crystal surfaces (Montel et al., 1981). Some insoluble material (tuft protein) also appears to be retained at prism boundaries at greatest concentrations near the enamel-dentine junction. Recent studies suggest that this may be cross linked, possibly a protection of the enamel dentine junction from Kallikrein 4. Its origins remain obscure but may comprise residual enamel peptides which have become crosslinked (Robinson et al., 1975; Robinson and Hudson, 2011). Whether this crosslinking occurs specifically during maturation is not known.

## Mineral uptake and crystal growth

**Figure 2:**
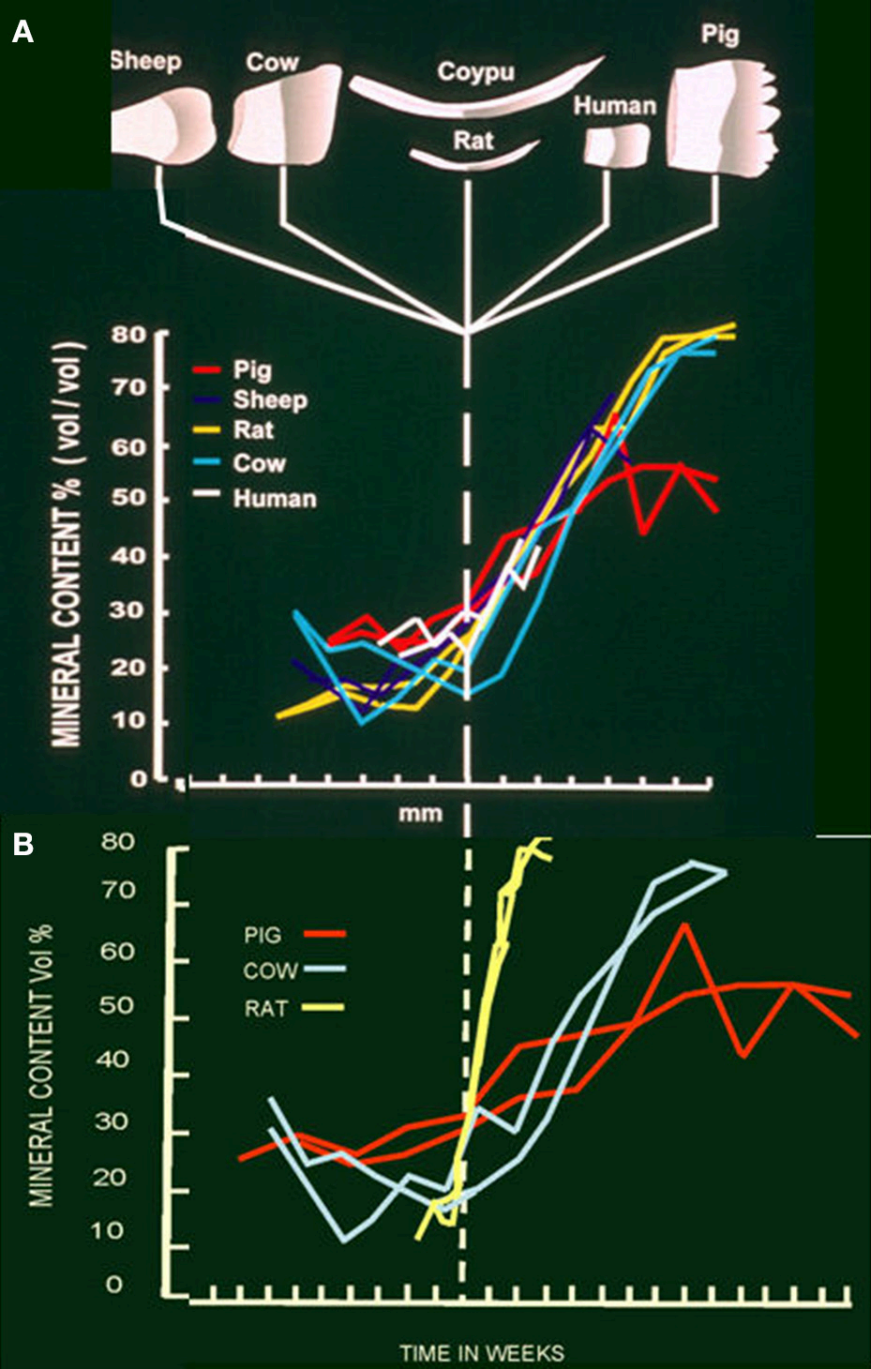
**(A)** Mineral content at each stage of development in developing teeth of sheep, cow, coypu, rat, human and pig. Steep increase in mineral content can be seen at the beginning of the maturation stage in each species. **(B)** Mineral content of developing teeth from pig, cow and rat in relation to duration of each stage. Maximum mineral content is achieved in about 2 weeks in the rat, 10 weeks in the cow and a minimum of about 13 weeks in the pig.

**Figure 3:**
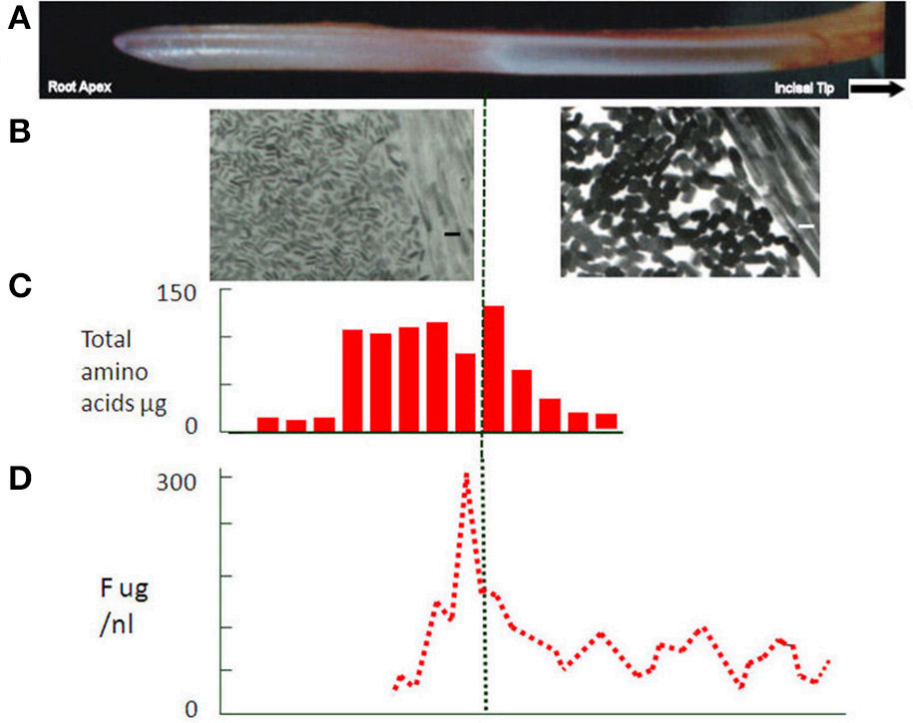
**(A)** Rat incisor showing secretory and white opaque maturation stages. In rat, enamel. **(B)** Electron micrographs of apatite crystals at secretory and maturation stages in the rat incisor at the same magnification (size bars = 30 nm). Much larger, growing crystals (~30 nm in width) can be seen in the maturation stage tissue. **(C)** Protein expressed as total amino acid content at each developmental stage of the rat incisor. Dramatic fall in protein content can be seen in the maturation stage. **(D)** Fluoride content at each stage of development of rat incisor enamel. Peak concentrations can be seen across the boundary between transition and maturation stages. The peak indicates some loss of free fluoride during the maturation stage.

As we move into the maturation stage proper, mineral ion uptake i.e., calcium and phosphate, increases and crystal growth in width and thickness accelerates (Figures 2A, 3B) (Nylen et al., 1963; Robinson et al., 1974; Hiller et al., 1975). This builds on some slower crystal growth in the enamel interior, which occurs during secretion.

Removal of matrix protein seems to be a prerequisite for crystal growth (Robinson et al., 1995) presumably, providing access of mineral ions to the growing crystals. Initial removal of proteins in interior, i.e., first formed, enamel would result in maturation growth accelerating from inside to outside. This would prevent sealing of the surface by rapid growth of surface crystals as ions enter from the ameloblast layer and probably reflects the sequence of protein removal from crystal surfaces.

Increase in crystal volume gradually displaces the fluid which had replaced the organic matrix and the tissue becomes much less hydrated, less porous and harder. The outer edge is the last part of the tissue to mature often erupting before completing leaving a white porous surface.

### Route of mineral ions

Mineral access to the enamel is extremely rapid (Robinson et al., 1974) and there has been considerable discussion as to whether this is an entirely active process. Recently evidence has been presented for specific ion transporters in the ameloblast membrane (Josephsen et al., 2010). The extent to which these are responsible for crystal growth is not yet determined. Active transport into the tissue may occur, regulating the access of ions and to some extent controlling the rate of crystal growth i.e., maturation. The simplest route, however, would be diffusion into the maturing tissue down a concentration gradient generated by growing crystals. Both mechanisms are not mutually exclusive.

## pH and maturation

There is an apparent drop in pH during maturation from about 7 to 6.5 (Sasaki, 1991). This has been attributed to growth of apatite crystals which would remove hydroxyl ions from the immediate environment. This prompted the suggestion that carbonic anhydrase, present during maturation in ruffle bordered cells, was needed to buffer the increasing amounts of acid produced as crystals grew (Smith et al., 2006). Since the pH does in fact fall, buffering is not complete. Alternatively, the duration of maturation, i.e., 2 weeks for the rat incisor, months in the cow and years in human teeth (Figure 2B) would seem to provide sufficient time for protons to diffuse out of the enamel and/or other buffers to diffuse in, phosphate, for example, is freely exchangeable with the circulation (Robinson et al., 1974) at this stage.

Since the pH drop has been specifically associated with ruffle ended ameloblasts, an alternative view is possible. It may be that protons are pumped into the enamel at this stage (Damkier et al., 2014) in order to generate a relatively low pH. The resulting protonation of crystal surfaces (Robinson et al., 2006) and perhaps residual peptides may facilitate removal of peptides from crystals thus promoting crystal growth. On the other hand such protonation could be a direct means of controlling the rate of crystal growth.

## Uptake of extraneous materials with implications for enamel dysplasias

It is clear that not only are calcium and phosphate taken up by the enamel during maturation but uptake of other materials also occurs. This is most likely facilitated by the high porosity and levels of hydration. Also penetration between ruffle ended ameloblast and lateral diffusion around smooth ended ameloblasts can occur. The close proximity of fenestrated capillaries to the stratum intermedium will in addition facilitate access to the circulation.

### Phosphate

This access was indicated by investigations using radioactive phosphate. Radioactive phosphate ions entered the enamel very shortly after injection. Two peaks of uptake were observed, one in early maturation which persisted and was presumably related to crystal growth. A second peak at the transition/maturation junction decreased, over a period of about 10 min probably reflecting blood levels of radioactive phosphate ions. It is presumed that this radioactive phosphate diffused out of the enamel as blood levels decreased (Robinson et al., 1974). This indicated that ions and possibly other materials could diffuse into and out of the enamel across the transition/maturation junction. A number of other components appear to behave in this way.

### Magnesium

Magnesium concentrations also show some indication of selective uptake at transition/early maturation followed by a loss. It is not clear what effect this may have, but magnesium can inhibit crystal growth, is often associated with fluoride (see below) and may affect enzyme activity (Hiller et al., 1975; Robinson et al., 1984).

### Fluoride

Selective fluoride uptake at this stage is of particular interest because of its effect on ameloblasts, enamel structure and dental caries. A peak of fluoride can be seen in transition and early maturation (Weatherell et al., 1975, 1977) (Figure 3D). Some of this fluoride is incorporated into the growing crystals but a substantial amount is clearly free and can, like phosphate, diffuse back out of the enamel. This free fluoride ion has become an important aspect of the development of fluorosis. Fluoride has been shown to affect F actin assembly in ameloblasts (Li et al., 2005). This is the likely explanation for the effect of fluoride on the modulation between ruffled and smooth ended maturation ameloblasts (denBesten et al., 1985) with a consequent effect on enamel maturation. Changes in the exposure time to ameloblasts providing calcium and phosphate or removing protein could lead to delayed crystal growth generating the porous immature appearance of fluorotic tissue.

With regard to fluoride incorporation into crystals at this stage, elevated fluoride can inhibit crystal growth by lowering surface energy. In addition, fluoride renders it more difficult to protonate (Robinson et al., 2006) the crystal surface possibly affecting removal of protective protein also affecting crystal growth rate.

### Carbonate

Interestingly, carbonate, high in the mineral during early development, does not increase during early maturation suggesting that it is likely a reflection of local metabolic CO_2_ production rather than serum bicarbonate. The high carbonate in the interior of mature enamel may, however, reflect the composition of the earliest formed mineral (Hiller et al., 1975) and the gradient of carbonate concentration seen in mature tissue (Weatherell et al., 1968). A similar argument can be made for the magnesium gradient in mature enamel (Robinson et al., 1981b).

### Albumin

Other materials can also be incorporated into the tissue at this stage. Of particular interest is albumin. Albumin at low levels has been reported to occur in secretory and especially maturing enamel (Strawich and Glimcher, 1989; Robinson et al., 1992). The latter may be due to the permeability of the ameloblast layer at this stage and the close proximity of fenestrated capillaries (Garant and Gillespie, 2005) as well as the porous nature of the enamel (Figure 1A). While this has been attributed by some to serum contamination, sufficient evidence exists that some albumin is present and in fact shows evidence of degradation due to enamel enzymes. One property of albumin pertinent to maturation is its ability to inhibit crystal growth (Garnett and Dieppe, 1990). This raised the possibility that leakage of albumin into maturing enamel, where crystals no longer are protected by endogenous matrix proteins, causes delayed maturation.

This may explain some types of idiopathic hypomaturation (white opaque hypoplasia) associated with, for example, physical trauma to the developing enamel (Tarján et al., 2002) or with some diseases of childhood. Under these circumstances hyperaemia can occur leading to leakage of albumin (and possibly other serum proteins) on to the growing crystals. Detection of albumin in such lesions has supported this view, for example, Kirkham et al. (2000).

### Tetracycline

The absence of defined lines in enamel after injection of tetracycline and its location at transition/maturation reflects the diffusion of tetracycline throughout the porous enamel of the early maturation stage (Hammarström, 1967). Uptake presumably occurs via the permeable ameloblast layer. This offers an explanation for tetracycline staining throughout enamel of children taking the antibiotic during the period of tooth development particularly maturation

## Summary

The maturation stage of enamel development comprises a secondary stage of mineral deposition during which apatite crystals, stage grow in width and thickness to replace fluid which had replaced the degraded protein matrix. The timescale for the maturation process is hugely variable dependent on species, from 2 weeks to several years. 90–95% of the tissue volume is finally occupied by apatite crystals. As a result of changes in the enamel organ and the porosity of maturing enamel extraneous materials can enter the tissue at this stage. Depending on the materials themselves and the duration of exposure, this can lead to delayed maturation and the eruption of white opaque dysplastic tissue. Enamel maturation is not only an important stage with regard to tissue development but also a very sensitive one in terms of hypomineralised enamel dysplasias.

### Conflict of interest statement

The author declares that the research was conducted in the absence of any commercial or financial relationships that could be construed as a potential conflict of interest.
